# Supplementary material for: Polycystin-1 Interacting Protein-1 (CU062) Interacts with the Ectodomain of Polycystin-1 (PC1)
Source: Cells. 2023 Aug 29;12(17):2166. doi: 10.3390/cells12172166 (PMC10487028; doi:10.3390/cells12172166)
Supplement: Supplementary file 1 [file cells-12-02166-s001.zip › cells-2534401-supplementary.pdf]

cells

MDPI

Article

Polycystin-1 Interacting Protein-1 (CU062) Interacts with the Ectodomain of Polycystin-1 (PC1)

Wendy A. Lea [1], Thomas Winklhofer [1], Lesya Zelenchuk [1], Madhulika Sharma [1], Jessica Rossol-Allison [2], Timothy A. Fields [3], Gail Reif [1], James P. Calvet [1], Jason L. Bakeberg [1], Darren P. Wallace [1] and Christopher J. Ward [1,*]

1 Department of Nephrology and Hypertension, The Jared Grantham Kidney Institute, University of Kansas Medical Center, Kansas City, KS 66160, USA
2 Promega Corporation, 2800 Woods Hollow Road, Madison, WI 53711, USA
3 Department of Pathology & Laboratory Medicine, University of Kansas Medical Center, 3901 Rainbow Blvd, Mail Stop 3062, Kansas City, KS 66160, USA
* Correspondence: cward6@kumc.edu

check for updates

Citation: Lea, W.A.; Winklhofer, T.; Zelenchuk, L.; Sharma, M.; Rossol-Allison, J.; Fields, T.A.; Reif, G.; Calvet, J.P.; Bakeberg, J.L.; Wallace, D.P. and Christopher J. Ward Polycystin-1 Interacting Protein-1 (CU062) Interacts with the Ectodomain of Polycystin-1 (PC1). *Cells* **2023**, *12*, 2166. https://doi.org/10.3390/cells12172166

Academic Editor: John Sayer

Received: 15 July 2023
Revised: 7 August 2023
Accepted: 14 August 2023
Published: 29 August 2023

Copyright: © 2023 by the authors. Licensee MDPI, Basel, Switzerland. This article is an open access article distributed under the terms and conditions of the Creative Commons Attribution (CC BY) license (https://creativecommons.org/licenses/by/4.0/).

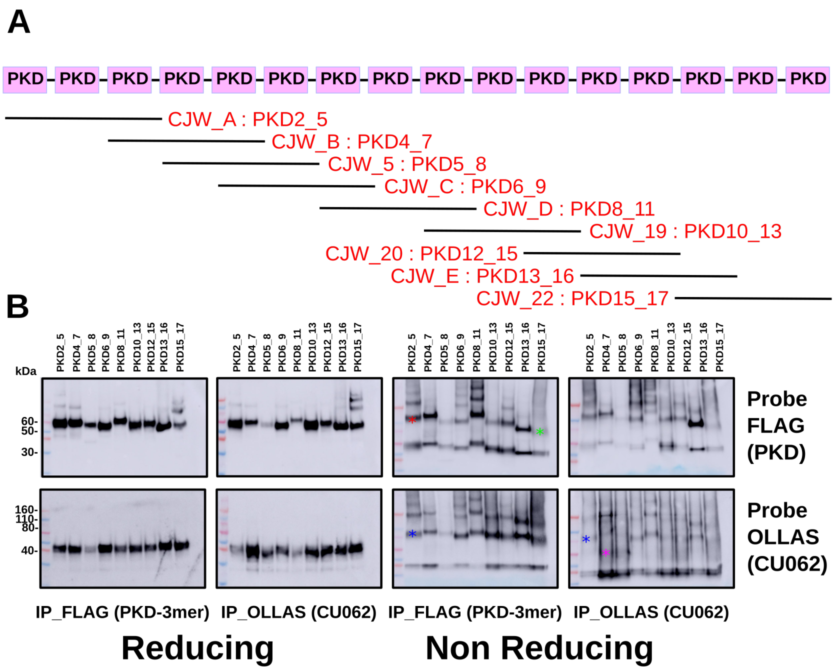

Figure S1. The CU062 protein interacts with trimer arrays of PKD domains. (**A**) Overlapping PKD domain 3mers were cloned downstream of a mouse PC1 signal peptide followed by a triple FLAG tag, a tobacco etch virus (TEV) proteinase site, and two Strept II tags (Table S1). (**B**) CU062 ::OLLAS and the appropriate PKD domain constructs were transfected into 293t cells for 10 h and IPs were performed (Table S2) [5]. Reducing gels: These showed that CU062 could reciprocally IP any of the PKD domain 3mers. Non-reducing gels: Top left and top right (probed PKD::FLAG); here, the PKD/PKD interaction was manifested in the first 8 IPs (red star), except in PKD 15_17, where the CU062/PKD repeat interaction was apparent (green star). Bottom left and right; here, the PKD/CU062 interaction was manifested (blue stars). The CU062/CU062 interaction is also visible in the lower right panel in lanes PKD−2_5, PKD−4_7, and PKD−5_8, (pink star). Controls with isotype control antibodies were always negative; see Figure 2 as an example.

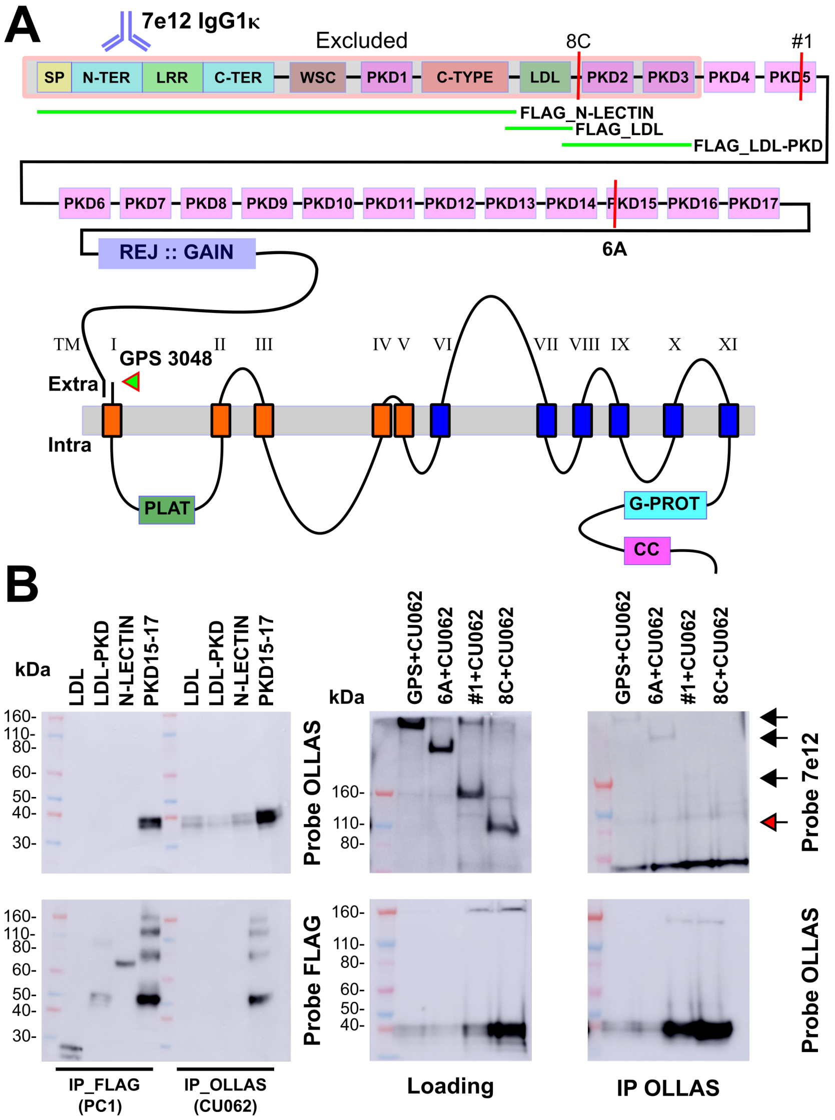

Figure S2. The CU062 protein interacts with the PKD domain array in PC1. (**A**) A map of PC1 showing the location of the epitope for the 7E12 antibody in the leucine-rich repeat, as well as the various constructs used. (**B**) The constructs N−terminal to the 4th PKD domain could not IP CU062. The transposon-truncated constructs 6A, #1, and the parent construct that extends to the native cleavage site at 3048 (GPS) could IP CU062 but the small clone 8C with a transposon-induced termination before the 2nd PKD domain could not IP CU062. Likewise, the small construct LDL−PKD (PKD II−III) could not IP CU062 ; see Table S1 and S2.

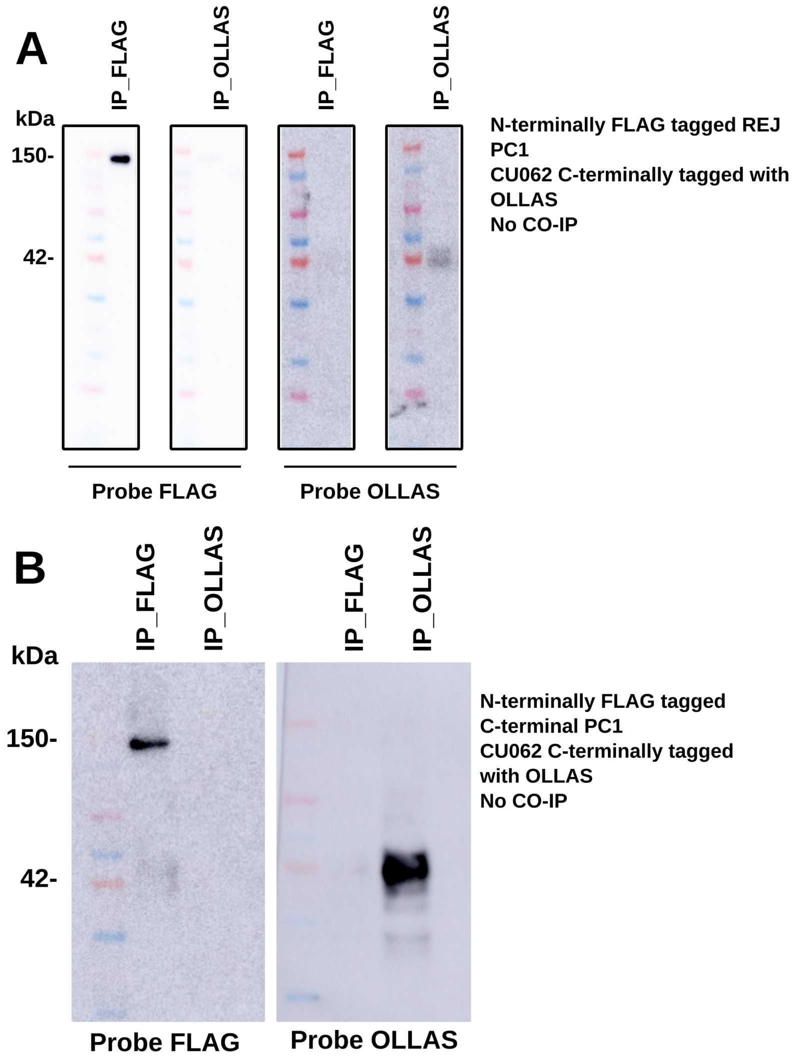

Figure S3. The CU062 protein fails to IP the receptor egg jelly (REJ) or C−terminus of PC1. (**A**) FLAG::REJ3 has an mPC1 signal peptide, followed by a triple FLAG tag, and then a TEV proteinase site followed by the PC1 REJ domain in its entirety (6403−9144 AQATVTV−VCLTRHL). CU062::OLLAS was co-expressed with FLAG::REJ3. FLAG IPed, the 150kDa FLAG::REJ3, but not the 42kDa CU062::OLLAS; OLLAS IPed CU062::OLLAS but not FLAG::REJ3; this implies that they do not interact. (**B**) FLAG::AH8 has an mPC1 signal peptide followed by a triple FLAG tag, and then the GPS cleavage site to the C−terminal end of PC1 (the entire 11 TM region); mPC1_SS_FLAG::TAFGASLF−NKVHPSST. CU062::OLLAS was co-expressed with AH8::FLAG. FLAG IPed, the 150 kDa AH8::FLAG, but not 42 kDa CU062::OLLAS. Conversely, OLLAS IPed CU062::OLLAS but not AH8::FLAG; this implies that they do not interact with each other, Table S1.

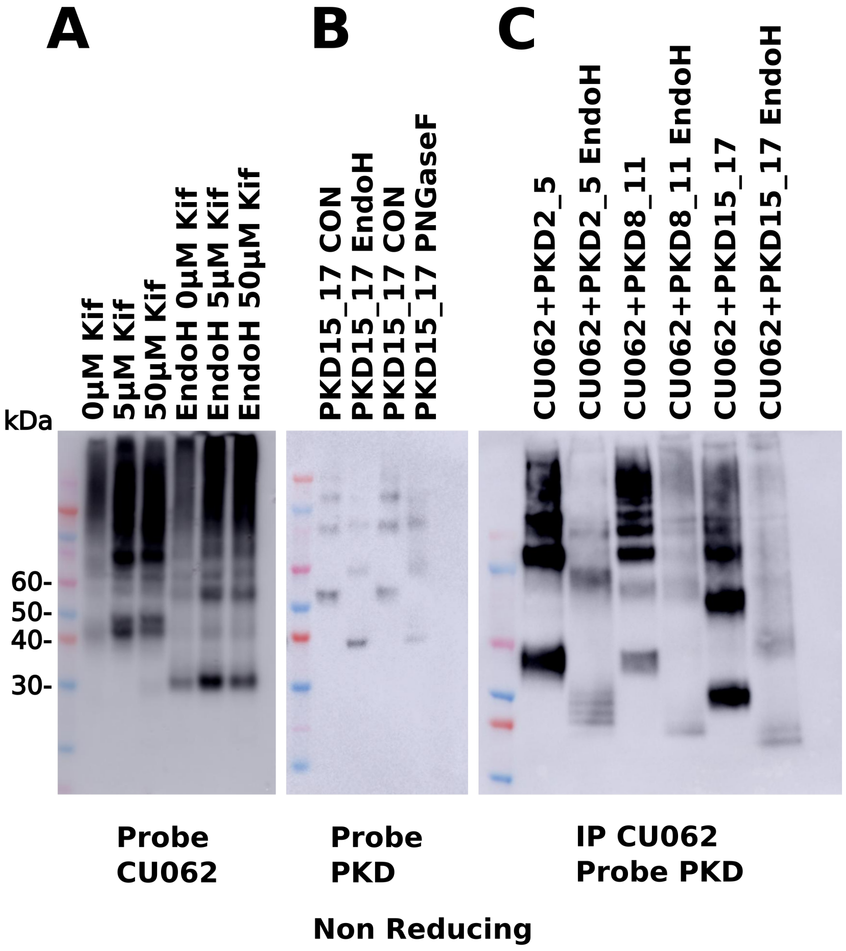

Figure S4. The PKD/PKD, CU062/CU062, and the PKD/CU062 interactions are carbohydrate-independent. (**A**) CU062:TAP (CU062:TAP ran at 30 kDa, deglycosylated due to the large TAP tag, 6984 Da) transiently transfected into 293t cells and was treated with the mannosidase I inhibitor kifunensine (to induce EndoH sensitivity). Lysates were treated with EndoH. Deglycosylation did not inhibit the oligomerization of the CU062 protein. (**B**) FLAG::PKD 15_17 transiently transfected into 293t cells and deglycosylated with EndoH or PNGase F. Deglycosylation did not inhibit the oligomerization of the PKD domain-containing proteins. (**C**) IPs of FLAG::PKD 2_5, FLAG::PKD8_11, FLAG::PKD15_17, and CU062::OLLAS using OLLAS to IP and FLAG to probe the blot. Lysates were treated with EndoH prior to IP. Again, deglycosylation does not inhibit the PKD/CU062 interaction; therefore, none of the interactions appears to be lectin-like.

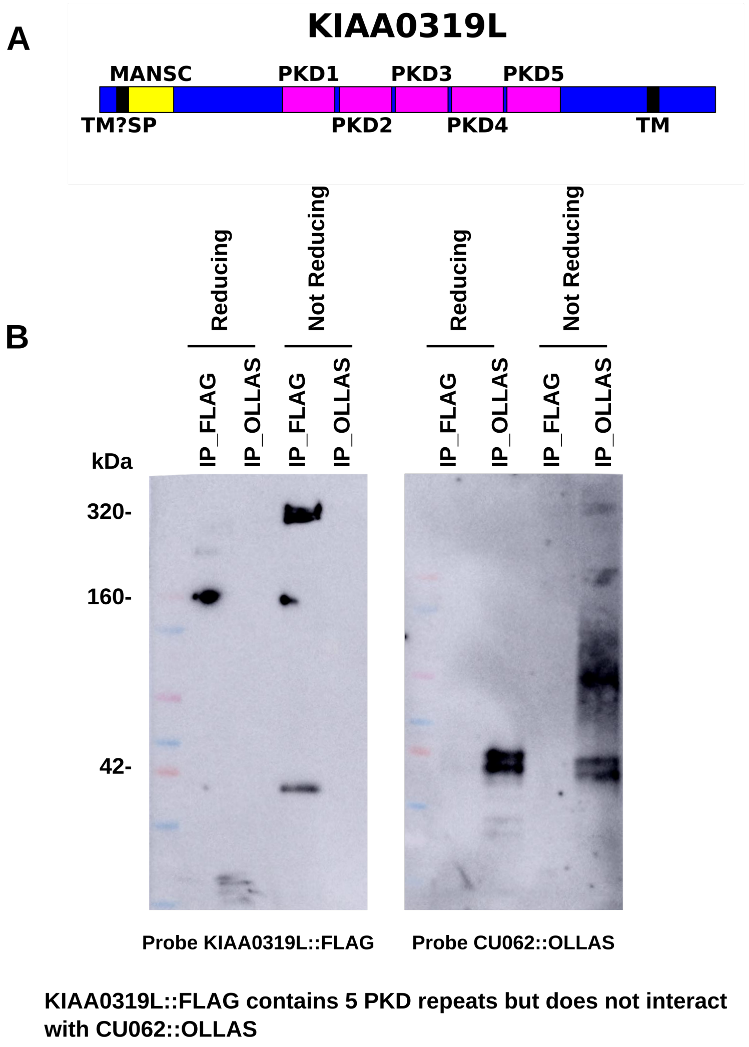

Figure S5. Full-length KIAA0319L does not interact with CU062, despite having five PKD domains. (**A**) KIAA0319L has a MANSC (motif at the N−terminus with seven cysteines) and five PKD domains. It has a membrane anchor or atypical signal peptide. (**B**) A full length KIAA0319L (NM_024874) (OriGene RC202618) with a C−terminal FLAG tag, co-expressed with CU062::OLLAS. FLAG antibody IPed KIAA0319L but not CU062, and OLLAS IPed CU062 but not KIAA0319L. KIAA0319L appears to dimerize under non-reducing conditions, perhaps via its PKD domains. KIAA0319L has five PKD domains but does not interact with CU062, showing that CU062 is specific for the PC1 PKD domains.

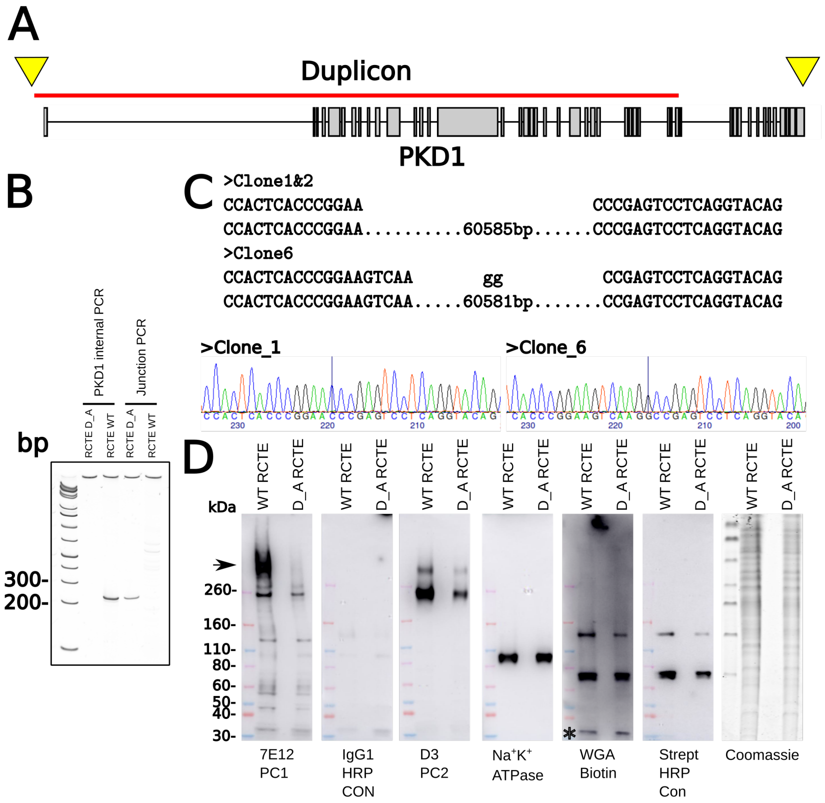

Figure S6. Targeting of the human *PKD1* gene and verification of mAb 7E12 (IgG1κ). (**A**) Two Cas12a CRISPRs were generated to the 3′ UTR of the *PKD1* gene and 5′ (centromeric) to the *PKD1* duplicon. The latter site was telomeric to the *RAB26* gene. (**B**) Genomic PCR with an internal PCR (exon-46) and PCR designed to amplify the junction fragment. (**C**) We succeeded in deleting the entire *PKD1* gene in RCTE cells, generating two alleles, deleting 60 585 bp and 60 581 bp of chromosome 16. This removed the entire *PKD1* gene in both cases. (**D**) Western blotting with 7E12 (IgG1κ) showed that the 460 kDa PC1 band (black arrow) was missing in the knockout clone, RCTE D_A. The peroxidase conjugate anti-IgG1::HRP goat anti-mouse secondary antibody failed to detect proteins. Anti-PC2 mAb (D3 IgG2bκ) detected the PC2 dimer and tetramer in WT and D_A but they were underrepresented in the knockout clone. The controls; Na^+K^+-ATPase, biotinylated wheat germ agglutinin (WGA), and streptavidin showed that the blots are equally loaded. The * shows a glycoprotein detected by WGA. Finally, a Coomassie Brilliant R-250 stained protein gel was used to confirm loading.

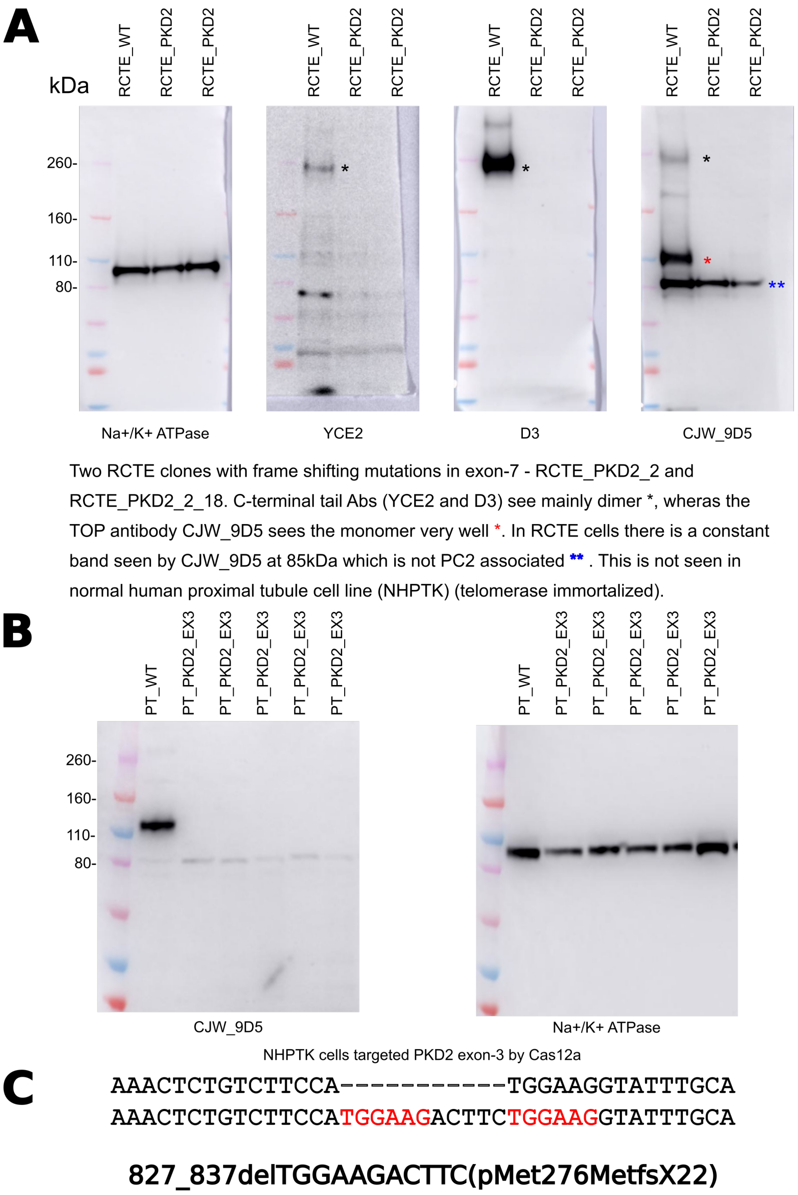

Figure S7. Targeting the human *PKD2* gene and verifying CJW_9D5 (IgG2bκ). (**A**) Four panels with WT RCTEs and RCTE clones with framing mutations in exon-7 of the human *PKD2* gene. The first panel shows that the lanes were equally loaded using $Na^{+}K^{+}$−ATPase as a control. The next two panels were probed with the commercially available anti-PC2 C−terminal tail antibodies, YCE2 (IgG2aκ AAs 687−754) and D3 (IgG2bκ AAs 689−968). Both mAbs recognized a dimeric PC2 band at 240 kDa (black star) and a tetrameric band at about 480 kDa that were absent in the *PKD2* mutant lanes. The new anti-TOP PC2 mAb CJW_9D5 (IgG2bκ AAs 242−468) recognized a monomer PC2 band at 115 kDa (red star) and a weaker dimeric band at 240 kDa, while these were absent in the *PKD2* mutant lanes. CJW_9D5 also recognized an 85 kDa band, which was not affected by the *PKD2* status and was non-specific in RCTE cells (double blue star). (**B**) Two panels with WT and five NHPTK clones with frame-shifting mutations in exon-3 of the *PKD2* gene (mutation described below). CJW_9D5 recognized only the 115 kDa band and was not observed in any of the *PKD2* disrupted subclones. There was no non-specific 85 kDa band in NHPTK cells, so this mAb was useful for imaging PT cells by IF. The right-hand panel was probed for $Na^{+}K^{+}$−ATPase to show equal loading, Table S3. (**C**) Cas12a-induced microhomology-mediated end joining generated a deletion in exon-3 of the *PKD2* gene in NHPTK cells. Red is the TGGAAG microhomology, which, upon CRISPR cutting and subsequent recombination, created an 11 bp deletion and frame-shifting mutation, abolishing all PC2 expression, as assessed by D3, YCE2, and CJW_9D5 antibodies, Table S3.

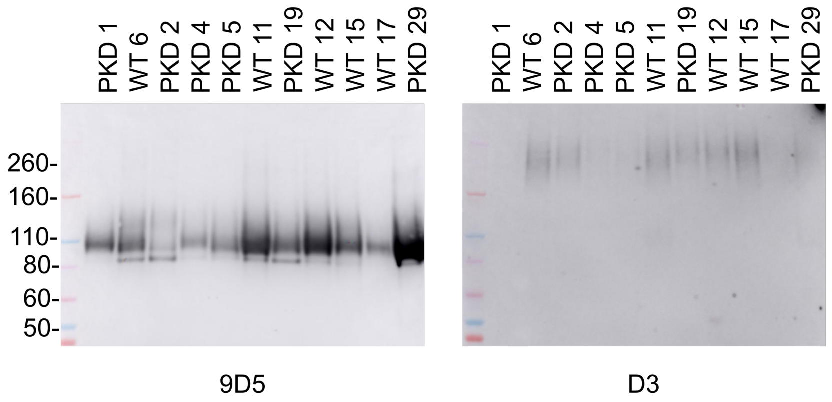

11 urine exosome samples probed with 9D5 and D3. 9D5 sees monomer D3 only sees dimer weakly. The 9D5 PC2 band is slightly smaller than the protein from cells

Figure S8. Western analysis of urinary PKD−ELVs with N−terminal and C−terminal anti−PC2 mAbs. Left panel: A random arrangement of five normal WT urine samples and six urine samples from individuals with defined *PKD1* mutations that were probed with CJW_9D5 (IgG2bκ). Note that PKD−ELV PC2 resolved at 107 kDa, slightly smaller than the cellular material at 115 kDa. This phenomenon is under investigation by the MS/MS analysis (there may be a cleavage event occurring at the C−terminal tail of PC2) [2]. Right panel: A duplicate of the left panel, except it was probed with D3 (IgG2bκ), a mAb to the C−terminal cytoplasmic tail of PC2. There was a faint smear at 240 kDa (PC2 dimer) and no material at 107 kDa (the PC2 monomer).

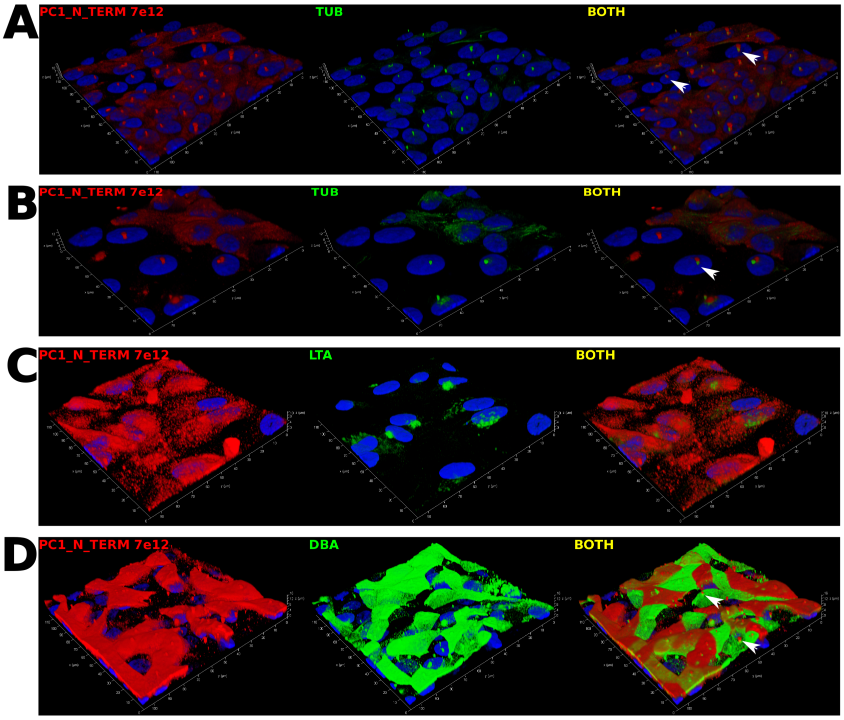

Figure S9. Localization of PC1 on confluent human primary kidney epithelial cells (HPREs). 3D confocal reconstructions of confluent and serum-starved (ciliated) HPRE cells: (**A**) HPRE cells stained with 7E12 (IgG1κ) to the LRR domain of PC1, extreme N−terminus, red. Primary cilia are detected with anti-acetylated α−tubulin (clone 6−11B−1, IgG2bκ), green. The anti-tubulin antibody was applied after the PC1 staining was performed to prevent colocalization artifacts due to the 'sticky' nature of the anti-tubulin mAb. The green cilia have 'blobs' of red PC1 applied to them, these are likely PKD−ELVs extracellularly attached to the primary cilia, as some cells have no internal PC1, yet have a small amount of PC1 on the primary cilia (white arrows). (**B**) A close-up of phenomenon, where cells that are negative for PC1 have primary cilia and are positive for PC1. (**C**) In order to determine the nature of the PC1-positive and -negative cells, we used the lectin *Lotus tetragonolobus* agglutinin (LTA), shown in green, as a PT marker. All LTA+ cells (Golgi stain) were PC1+, displayed in red. (**D**) To detect CD cells, we used the lectin *Dolichos biflorus* agglutinin (DBA), shown in green. Most DBA+ cells were PC1− in the cell body; however, some had PC1+ extracellular vesicles associated with their primary cilia (white arrows). This suggests that PC1+ PT cells can shed PC1+ PKD−ELVs, which can interact with the primary cilia of CD cells [4].

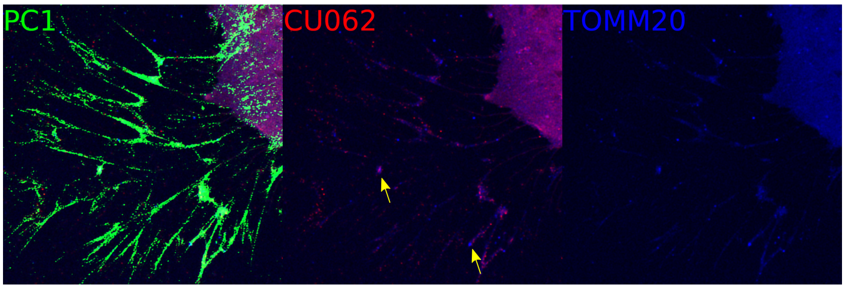

Figure S10. Treatment of HPRE cells with 2 µM CCCP for 9 hours induces mitocytosis. PT cells were treated with 2 µM CCCP for 9 h. The cells leave a trail of RF across the fibronectin and were PC1+ (7E12), shown in green. Migrasomes were also CU062 + (CJW_2D11), shown in red. Many migrasomes contained TOMM20+ material, which were the remnants of senescent mitochondria, represented by yellow arrows [3].

Table S1. PC1 and PC2 DNA constructs used in the present study.

Name	Range	AA Range	Domains	Apparent Mwt (kDa) Glycosylated
CJW_A	2191 − 3060	PAPGH − NMRMQ	Contains PKD 2, 3, and 4 repeats	60
CJW_B	2785 − 3648	EEPIC − LRGLS	Contains PKD 4, 5, and 6 repeats	60
CJW_5	3034 − 3909	TVERM − VEPAA	Contains PKD 5, 6, and 7 repeats	57
CJW_C	3367 − 4152	RASLP − VGNVT	Contains PKD 6, 7 and 8 repeats	55
CJW_D	3877 − 4656	VLEVL − VRGLV	Contains PKD 8, 9, and 10 repeats	62
CJW_19	4372 − 5160	ALVEV − EPVGW	Contains PKD 10, 11, and 12 repeats	60
CJW_20	4894 − 5661	IEGLQ − AEEPI	Contains PKD 12, 13, and 14 repeats	60
CJW_E	5143 − 5925	VEPVG − VSGLQ	Contains PKD 13, 14, and 15 repeats	57
CJW_22	5632 − 6522	TYNLT − EAHVD	Contains PKD 15, 16, and 17 repeats	57
FLAG::REJ3	6403 − 9144	AQATV − LTRHL	Contains PKD REJ domain	150
GPS::FLAG	1 − 9144	MPPAA−LTRHL	SP to GPS cleavage then TRIPLE FLAG	>300
Exon_20::FLAG	1−7863	MPPAA−TVLNE	SP to mid-REJ domain	300
6A	1 − 5787	MPPAA−GANPE	SP to PKD 15 middle	260
#1	1 − 3228	MPPAA−VLASN	SP to PKD 5 end	160
8C	1 − 2268	MPPAA−PHLPA	SP to PKD 2 begin	110
FLAG::AH8	9145− 12909	TAFGA−HPSST	N−terminal triple FLAG TMI−XI	150
TagGFP2::PC2	1 − 968	MVNSS−SNVHV	TagGFP2::Full PC2 ORF	150

Table S2. CU062 DNA constructs used in the present study.

Name	Range	Tag	Domains	Apparent Mwt (kDa) glycosylated
CU062::V5	All 219 AAs	1X V5	DUF4571	40 kDa−42 kDa
CU062::OLLAS	All 219 AAs	2X OLLAS	DUF4571	42 kDa
CU062::TAP	All 219 AAs	1X TEV 2X Strept II 1X tFLAG	DUF4571	45 kDa

Table S3. Antibodies used in the present study.

Name	Antigen	Host	Isotype	Source
7E12	LRR PC1	Mouse anti-human	IgG1κ	[6]
Ab11	TIG1−3 Fibrocystin	Mouse anti-human	IgG1κ	[1]
Ab18	TIG1−3 Fibrocystin	Mouse anti-human	IgG2aκ	[1]
CJW_2D11	CU062	Mouse anti-human	IgAκ	This publication
CJW_6F3	CU062	Mouse anti-human	IgG1κ	This publication
CJW_9D5	PC2	Mouse anti-human	IgG2bκ	This publication
D3	PC2	Mouse anti-human	IgG2bκ	sc-28331
YCE2	PC2	Mouse anti-human	IgG2aκ	sc-47734
6-11B-1	acetylated α-tubulin	Mouse anti-*Strongylocentrotus*	IgG2bκ	T7451 (Sigma)
H-3	Na^+/K^+−ATPase−α1	Mouse anti-human	IgG2bκ	sc-48345
Talin C−9	Talin AAs 1−300	Mouse anti-human	IgG3κ	sc-365875
3G5	TOMM20	Rabbit anti-human	IgG (mAb)	24859 (Thermo Fisher)
D6F6	α−actinin	Rabbit anti-human	IgG (mAb)	#6487 (Cell Signaling)
NBP1:59438	TSPAN4	Rabbit anti-human	IgG (Polyclonal)	(Novus Biologicals)
17711-1-AP	ARL13B	Rabbit anti-human	IgG (Polyclonal)	(Proteintech)

References

1. Hogan, M.C.; Manganelli, L.; Woollard, J.R.; Masyuk, A.I.; Masyuk, T.V.; Tammachote, R.; Huang, B.Q.; Leontovich, A.A.; Beito, T.G.; Madden, B.J.; et al. Characterization of PKD Protein-Positive Exosome-Like Vesicles. *J. Am. Soc. Nephrol.* **2009**. https://doi.org/10.1681/ASN.2008060564.
2. Lea, W.A.; McGreal, K.; Sharma, M.; Parnell, S.C.; Zelenchuk, L.; Charlesworth, M.C.; Madden, B.J.; Johnson, K.L.; McCormick, D.J.; Hogan, M.C.; et al. Analysis of the polycystin complex (PCC) in human urinary exosome-like vesicles (ELVs). *Sci. Rep.* **2020**, *10*, 1500.
3. Jiao, H.; Jiang, D.; Hu, X.; Du, W.; Ji, L.; Yang, Y.; Li, X.; Sho, T.; Wang, X.; Li, Y.; et al. Mitocytosis, a migrasome-mediated mitochondrial quality-control process. *Cell* **2021**, *184*, 2896−2910.

4. Bakeberg, J.L.; Tammachote, R.; Woollard, J.R.; Hogan, M.C.; Tuan, H.F.; Li, M.; van Deursen, J.M.; Wu, Y.; Huang, B.Q.; Torres, V.E.; et al. Epitope-tagged Pkhd1 tracks the processing, secretion, and localization of fibrocystin. *J. Am. Soc. Nephrol.* **2011**, *22*, 2266−2277. https://doi.org/10.1681/ASN.2010111173.
5. Park, S.H.; Cheong, C.; Idoyaga, J.; Kim, J.Y.; Choi, J.H.; Do, Y.; Lee, H.; Jo, J.H.; Oh, Y.S.; Im, W.; et al. Generation and application of new rat monoclonal antibodies against synthetic FLAG and OLLAS tags for improved immunodetection. *J. Immunol. Methods* **2008**, *331*, 27−38.
6. Ong, A.C.; Harris, P.C.; Davies, D.R.; Pritchard, L.; Rossetti, S.; Biddolph, S.; Vaux, D.J.; Migone, N.; Ward, C.J. Polycystin-1 expression in PKD1, early-onset PKD1, and TSC2/PKD1 cystic tissue. *Kidney Int.* **1999**, *56*, 1324−1333.

Disclaimer/Publisher's Note: The statements, opinions and data contained in all publications are solely those of the individual author(s) and contributor(s) and not of MDPI and/or the editor(s). MDPI and/or the editor(s) disclaim responsibility for any injury to people or property resulting from any ideas, methods, instructions or products referred to in the content.
